# Anterior Segment OCT in Fulminant *Pseudomonas aeruginosa* Corneal Ulcer with Stromal Melting Requiring Emergency Penetrating Keratoplasty

**DOI:** 10.3390/diagnostics16081189

**Published:** 2026-04-16

**Authors:** Wojciech Luboń, Monika Sarnat-Kucharczyk, Mariola Dorecka

**Affiliations:** 1 Department of Ophthalmology, Faculty of Medical Sciences, Medical University of Silesia, 40-514 Katowice, Poland; msarnat@sum.edu.pl (M.S.-K.); mdorecka@sum.edu.pl (M.D.); 2 Department of Ophthalmology, Professor K. Gibiński University Clinical Center, Medical University of Silesia, 40-514 Katowice, Poland

**Keywords:** infectious keratitis, in vivo confocal microscopy, corneal ulcer, penetrating keratoplasty, anterior segment optical coherence tomography, slit-lamp imaging, corneal perforation, *Pseudomonas aeruginosa*, stromal melting

## Abstract

Rapidly progressive infectious keratitis may involve the anterior uveal tract and lead to anterior segment inflammation, resulting in severe structural damage of the cornea and potentially causing corneal perforation or endophthalmitis if not promptly treated. We report the case of a 63-year-old male admitted to the Emergency Ophthalmology Department of the University Clinical Center in Katowice, Poland, with a rapidly progressive corneal ulcer of the left eye that had not responded to two weeks of outpatient topical antibiotic therapy. The condition developed after ocular trauma sustained while chopping wood. At presentation, visual acuity was limited to light perception with preserved projection. Multimodal imaging, including slit-lamp examination, anterior segment optical coherence tomography (AS-OCT), and in vivo confocal microscopy, revealed extensive corneal ulceration with severe stromal destruction, progressive corneal melting, and marked anterior segment inflammation, with an imminent risk of perforation. Microbiological cultures identified *Pseudomonas aeruginosa.* Despite intensive empiric topical antimicrobial therapy targeting both bacterial infection and a possible fungal component related to trauma with organic material, rapid clinical deterioration necessitated emergency therapeutic penetrating keratoplasty (PK). The procedure resulted in rapid resolution of inflammation and improvement in visual acuity, with best-corrected visual acuity (BCVA) reaching 0.3 logMAR during follow-up. At the three-month follow-up, the corneal graft remained clear with stable visual acuity and no recurrence of infection. The patient remains under regular long-term follow-up, with ongoing monitoring of graft clarity, intraocular pressure (IOP), and visual function. This case differs from routine presentations of infectious keratitis by demonstrating exceptionally rapid stromal melting despite promptly initiated empiric topical therapy. Multimodal imaging, particularly AS-OCT provided clinically meaningful information by revealing structural instability and an imminent risk of perforation not fully appreciable on slit-lamp examination, thereby supporting timely urgent keratoplasty. These findings highlight the practical diagnostic value of imaging-based assessment in advanced infectious keratitis and underscore its role in guiding surgical decision-making in eyes at high risk of corneal perforation.

**Figure 1:**
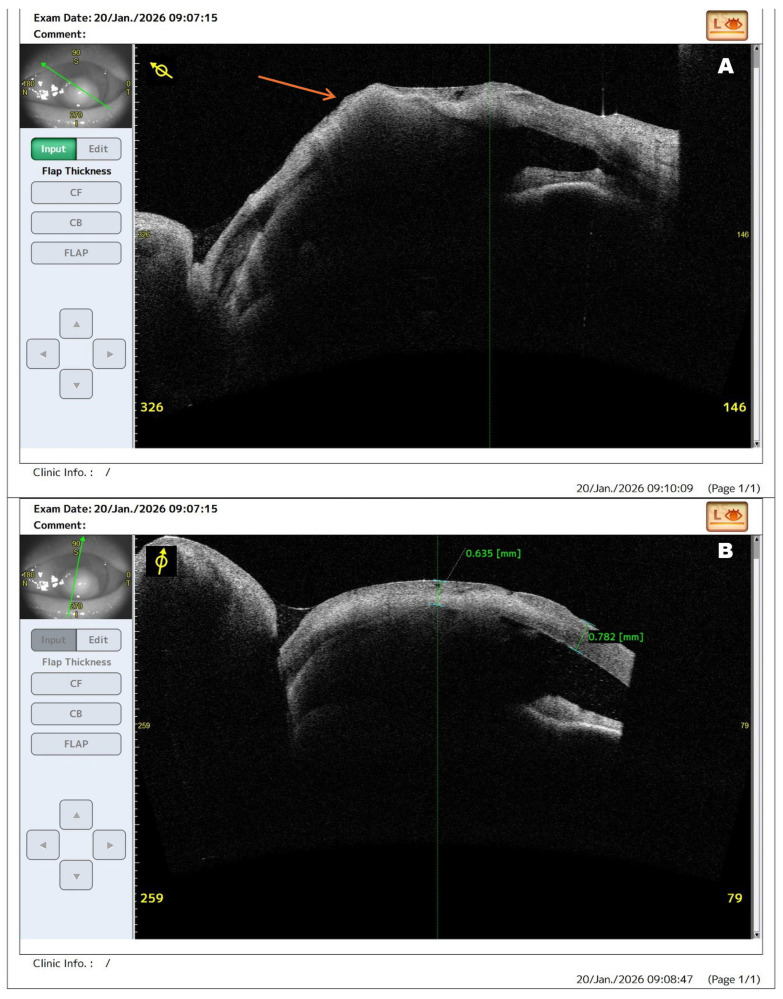
Anterior segment optical coherence tomography (AS-OCT) images of the left eye obtained at admission demonstrating extensive corneal structural damage in fulminant infectious keratitis. Infectious keratitis remains an ophthalmic emergency because rapid stromal destruction may lead to corneal perforation, endophthalmitis, and permanent visual loss if treatment is delayed. In advanced cases, multimodal imaging may provide clinically relevant information beyond slit-lamp biomicroscopy by enabling more precise assessment of stromal involvement, tissue integrity, and risk of impending perforation [1,2]. Therapeutic penetrating keratoplasty (PK) is reserved for severe cases with progressive stromal melting, uncontrolled infection, or threatened perforation despite medical treatment [3,4,5,6]. The present report is of particular interest because it demonstrates how AS-OCT, supported by slit-lamp examination and in vivo confocal microscopy, documented rapidly evolving structural instability in fulminant *Pseudomonas aeruginosa* keratitis and directly supported the decision for urgent surgical intervention. A 63-year-old male presented with rapidly progressive visual deterioration and severe ocular pain in the left eye following ocular trauma sustained while chopping wood two weeks before admission. Despite outpatient topical antibiotic therapy, the condition worsened, and visual acuity was limited to light perception with preserved projection. AS-OCT cross-section (**A**) demonstrates pronounced stromal edema, irregular anterior surface contour, and focal stromal thinning. The arrow indicates the area of maximal corneal melting corresponding to the ulcer and the site of impending perforation. The normal layered architecture of the cornea is largely lost, with hyperreflective inflammatory infiltrates within the stroma. Scan (**B**), obtained in a different meridian, reveals severe stromal disorganization with hyperreflective deposits extending toward the endothelial layer. These findings indicate advanced stromal melting and a high risk of imminent perforation. In the present case, AS-OCT enabled visualization of structural instability not fully appreciable on slit-lamp examination, thereby supporting timely surgical decision-making. AS-OCT provides valuable structural information in infectious keratitis by enabling assessment of corneal thickness and tissue integrity and helping identify eyes at high risk of perforation requiring urgent intervention [1,2].

**Figure 2:**
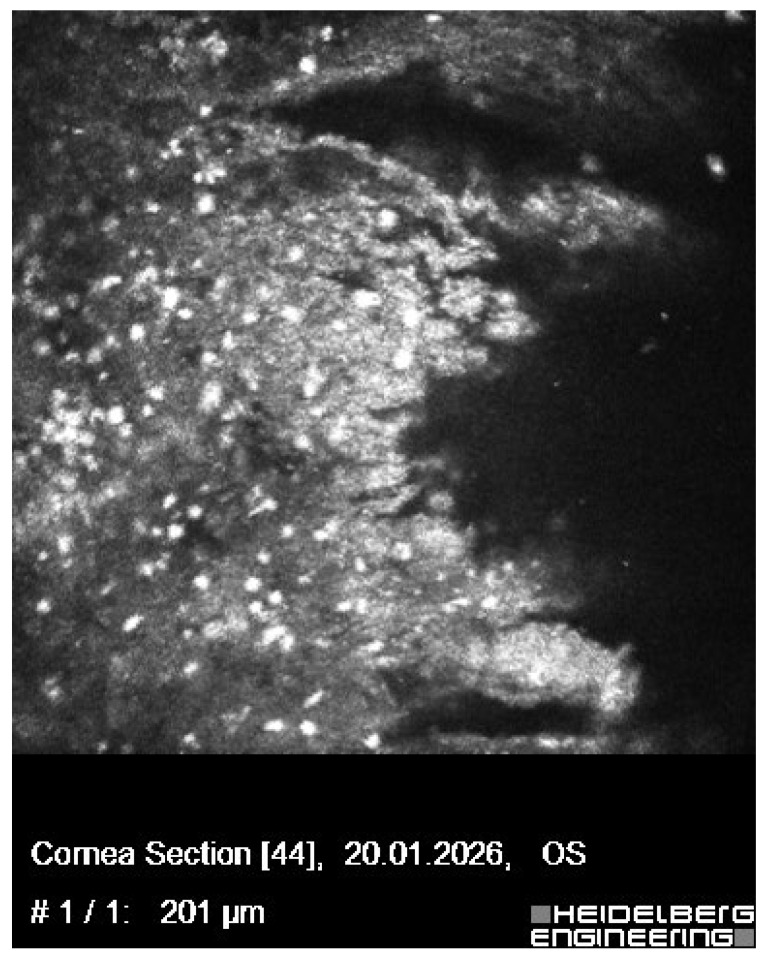
In vivo confocal microscopy image of the left cornea obtained at presentation, demonstrating microstructural features of active infectious keratitis. The image shows numerous hyperreflective cellular elements within the anterior corneal stroma, consistent with inflammatory cell infiltration. The normal stromal architecture is markedly disrupted, with loss of the typical keratocyte pattern and disorganization of the extracellular matrix at the level of the ulcer. These findings are indicative of active inflammation and stromal involvement in severe infectious keratitis. In vivo confocal microscopy enables high-resolution, real-time visualization of corneal microstructure and provides valuable adjunctive information in the diagnosis and assessment of infectious keratitis by detecting inflammatory cell infiltration and structural alterations not visible on slit-lamp examination [1].

**Figure 3:**
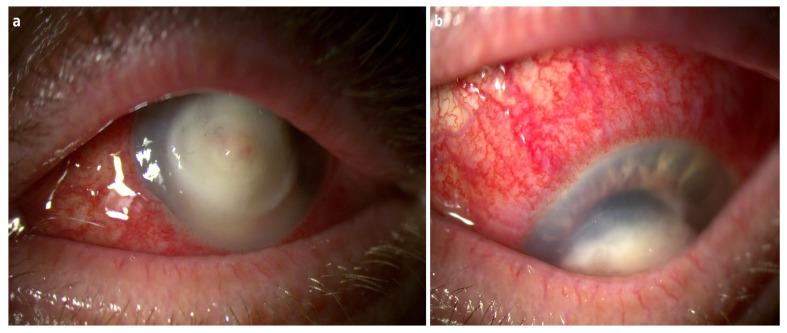
Slit-lamp photographs of the left eye obtained at presentation demonstrating advanced infectious keratitis with severe corneal ulceration and pronounced inflammatory involvement of the anterior segment. High-magnification slit-lamp image (**a**) showing a large central corneal ulcer with dense whitish stromal infiltration and indistinct margins. Marked corneal edema and stromal opacification are present, consistent with severe infectious keratitis and progressive stromal degradation. Image (**b**) obtained during downward gaze demonstrates extensive conjunctival and deep episcleral injection with pronounced dilation of episcleral vessels, indicating severe ocular surface inflammation and marked anterior segment inflammatory reaction. The coexistence of corneal ulceration, intense ocular hyperemia, and intraocular inflammatory response reflects the aggressive clinical course of the infection. Microbiological evaluation, including corneal scraping and conjunctival sac swabs, confirmed abundant growth (+++) of *Pseudomonas aeruginosa*. The patient was immunocompetent, with no history of systemic disease or immunosuppressive therapy. Antimicrobial susceptibility testing demonstrated sensitivity to ciprofloxacin, levofloxacin, gentamicin, and amikacin, with borderline susceptibility to piperacillin and tobramycin. Empiric topical therapy was initiated upon admission and included broad antimicrobial coverage tailored to the clinical context of trauma with organic material, in which a mixed bacterial–fungal infection could not be excluded. Fortified gentamicin 1.4% and topical moxifloxacin were administered alternately every hour, while topical voriconazole was also given every 2 h and chlorhexidine five times daily as adjunctive empiric antifungal coverage. Despite this intensive empiric topical regimen, rapid clinical deterioration was observed over the following 48 h, with progressive stromal melting and increasing risk of perforation. Given the fulminant course and lack of response to treatment, urgent therapeutic PK was performed to remove infected tissue and preserve globe integrity. Such an approach is consistent with current clinical practice in advanced infectious keratitis with progressive stromal degradation or impending perforation [3,6,7,8].

**Figure 4:**
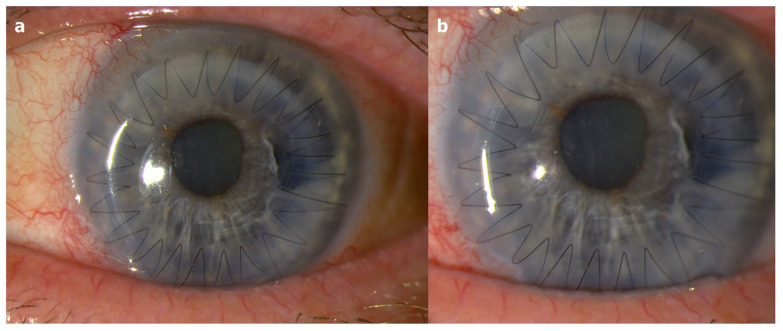
Slit-lamp photographs of the left eye obtained after emergency therapeutic PK demonstrating restoration of corneal integrity and resolution of the inflammatory process. Postoperative slit-lamp images (**a**,**b**) show a well-centered PK graft with a diameter of 8.25 mm secured with a continuous 10-0 nylon suture. The graft appears clear with good apposition to the recipient cornea and no signs of residual stromal infiltration. The anterior chamber is quiet and well formed, and the previously observed inflammatory reaction has resolved. At follow-up examination, best-corrected visual acuity (BCVA) improved to 0.3 logMAR. These findings confirm successful surgical management of fulminant infectious keratitis with restoration of corneal integrity and functional visual improvement. Therapeutic PK remains an important treatment option in advanced infectious keratitis with progressive stromal melting or imminent corneal perforation when medical therapy fails to control the infection [3,4,6].

**Figure 5:**
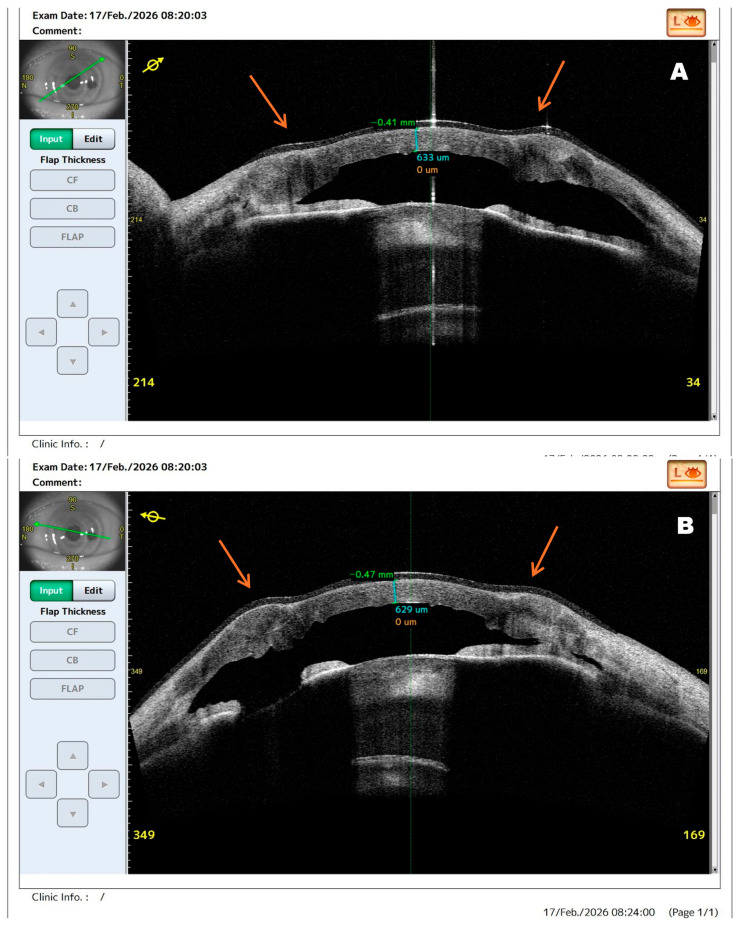
AS-OCT images obtained during postoperative follow-up demonstrating restoration of corneal integrity after emergency therapeutic PK. Cross-sectional scans (**A**,**B**) reveal a well-integrated corneal graft with restored corneal thickness and regular anterior surface curvature. The anterior chamber is re-formed and well defined; however, it appears relatively shallow, with focal narrowing of the anterior chamber angle visible in selected regions on AS-OCT. This configuration is likely multifactorial, potentially reflecting postoperative corneal edema with increased corneal thickness, as well as lens-related anterior segment crowding. Arrows indicate the donor–recipient junction corresponding to the graft–host interface and the location of the continuous 10-0 nylon corneal suture. The interface appears well apposed without evidence of residual stromal infiltration or structural instability. Compared with preoperative findings (Figure 1), postoperative AS-OCT confirms structural stabilization of the cornea and successful graft integration. Early surgical intervention at a critical stage of rapidly progressive infectious keratitis likely prevented further intraocular spread of infection. Intraocular pressure (IOP) in the postoperative period remained stable at 12 mmHg, as measured by Goldmann applanation tonometry. Therapeutic PK performed before corneal perforation may reduce the risk of severe complications, including endophthalmitis and potential loss of the eye, while enabling restoration of corneal structure and visual function [3,4]. Previous reports have highlighted the role of AS-OCT and in vivo confocal microscopy in the evaluation of infectious keratitis, particularly for assessing stromal involvement and inflammatory changes [1,2]. Therapeutic PK remains an established option in advanced infection with progressive stromal melting or impending perforation when medical therapy is insufficient [3,4]. Of particular interest to cornea specialists, this case illustrates how AS-OCT may help define the optimal window for urgent PK by detecting structural decompensation before frank perforation, thereby enabling earlier and more controlled surgical management of fulminant infectious keratitis.

## Data Availability

The original contributions presented in this study are included in the article. Further inquiries can be directed to the corresponding author.

## Abbreviations

The following abbreviations are used in this manuscript:

AS-OCT	Anterior Segment Optical Coherence Tomography
BCVA	Best-corrected Visual Acuity
IOP	Intraocular pressure
PK	Penetrating Keratoplasty
